# Supplementary material for: Effects of long-term and high-dose administration of glucocorticoids on the cranial cruciate ligament in healthy beagle dogs
Source: PLoS One. 2022 Jan 21;17(1):e0262206. doi: 10.1371/journal.pone.0262206 (PMC8782538; doi:10.1371/journal.pone.0262206)
Supplement: Attachment — Submitted filename: PONE-D-21-25951.pdf [file pone.0262206.s002.pdf]

PLOS ONE

Effects of long-term and high-dose administration of glucocorticoids on the cranial cruciate ligament in healthy beagle dogs

--Manuscript Draft--

Manuscript Number:	PONE-D-21-25951
Article Type:	Research Article
Full Title:	Effects of long-term and high-dose administration of glucocorticoids on the cranial cruciate ligament in healthy beagle dogs
Short Title:	Glucocorticoid effects on cranial cruciate ligament in dogs
Corresponding Author:	Masakazu Shimada<br>Nippon Veterinary and Life Science University<br>Musashino-shi, Tokyo JAPAN
Keywords:	cranial cruciate ligament; dog; glucocorticoids
Abstract:	This study aimed to determine the effects of long-term and high-dose administration of glucocorticoids (GCs) on the histological and mechanical properties of the cranial cruciate ligament (CrCL) in healthy beagle dogs. A synthetic corticosteroid at 2 mg/kg every 12 h was administered for three months in nine dogs (18 CrCLs) (GC group). Twenty CrCLs from 12 healthy male beagles were used as the normal control (control group). CrCLs were histologically examined (n=12 in the GC group and n=14 in the control group) using hematoxylin-eosin, Alcian-Blue, and Elastica-Eosin stains, and immunohistological staining of type 1 collagen and elastin. An additional 12 CrCLs were mechanically tested (n=6 in the GC group and n=6 in the control groups) to determine failure pattern, maximum tensile strength, maximum stress, elastic modulus, and stress and strain at the transition point. The histological examination revealed a significant increase in interfascicular area and fibrillar disorientation at the tibial attachment in both groups. The ratios of mucopolysaccharide-positive area and positive areas of elastic fibers were significantly higher in the control group than in the GC group. The biomechanical examination demonstrated significantly lower stress at the transition point in the GC group than in the control group. The results of the present study indicate that high-dose corticosteroid may affect metabolism, such as the production of mucopolysaccharides and elastic fibers, although the effect on type 1 collagen production is small. These changes of the extracellular matrix had a small effect on the strength of the ligament. This study suggested that the ligamentous changes associated with GC are different from the degeneration observed in spontaneous canine CrCL disease.
Order of Authors:	Masakazu Shimada
	Koki Wada
	Sachiyo Tanaka
	Sawako Murakami
	Nobuo Kanno
	Kei Hayashi
	Yasushi Hara
Additional Information:	
**Question**	**Response**
**Financial Disclosure**<br><br>Enter a financial disclosure statement that describes the sources of funding for the work included in this submission. Review the submission guidelines for detailed	The author (Y.H) was funded by a Grant-in-Aid for Scientific Research from the Japan Society for the Promotion of Science (Grant number: 17K08114, URL:https://www.jsps.go.jp/english/e-grants/). The creation of the model dogs in this study was supported by the Grant. The funders had no role in study design, data collection and analysis, decision to publish, or preparation of the manuscript.

Powered by Editorial Manager® and ProduXion Manager® from Aries Systems Corporation

requirements. View published research articles from *PLOS ONE* for specific examples.<br><br>This statement is required for submission and **will appear in the published article** if the submission is accepted. Please make sure it is accurate.<br><br>**Unfunded studies**<br>Enter: *The author(s) received no specific funding for this work.*<br><br>**Funded studies**<br>Enter a statement with the following details:<br>• Initials of the authors who received each award<br>• Grant numbers awarded to each author<br>• The full name of each funder<br>• URL of each funder website<br>• Did the sponsors or funders play any role in the study design, data collection and analysis, decision to publish, or preparation of the manuscript?<br>• **NO** - Include this sentence at the end of your statement: *The funders had no role in study design, data collection and analysis, decision to publish, or preparation of the manuscript.*<br>• **YES** - Specify the role(s) played.<br><br>* typeset	
**Competing Interests**<br><br>Use the instructions below to enter a competing interest statement for this submission. On behalf of all authors, disclose any competing interests that could be perceived to bias this work—acknowledging all financial support and any other relevant financial or non-financial competing interests.<br><br>This statement is required for submission and **will appear in the published article** if the submission is accepted. Please make sure it is accurate and that any funding sources listed in your Funding Information later in the submission form are also declared in your Financial Disclosure	The authors have declared that no competing interests exist.

statement.<br><br>View published research articles from *PLOS ONE* for specific examples.<br><br>**NO authors have competing interests**<br><br>Enter: *The authors have declared that no competing interests exist.*<br><br>**Authors with competing interests**<br><br>Enter competing interest details beginning with this statement:<br><br>*I have read the journal's policy and the authors of this manuscript have the following competing interests: [insert competing interests here]*<br><br>* typeset	
**Ethics Statement**<br><br>Enter an ethics statement for this submission. This statement is required if the study involved:<br><br>• Human participants<br>• Human specimens or tissue<br>• Vertebrate animals or cephalopods<br>• Vertebrate embryos or tissues<br>• Field research<br><br>Write "N/A" if the submission does not require an ethics statement.<br><br>General guidance is provided below. Consult the submission guidelines for detailed instructions. **Make sure that all information entered here is included in the Methods section of the manuscript.**	This study was approved by the Institutional Bioethics Committee of the Experimental Animal Committee(approval number: 2019S-60 and 2020S-25). Also, the experimental model was created for other unrelated approved studies (approval number: 2019S-72, 29S-37), and normal beagles were euthanized for other unrelated approved studies (approval number: 2019J-29, 30K-9) were used as the control group.

Powered by Editorial Manager® and ProduXion Manager® from Aries Systems Corporation

**Format for specific study types**<br><br>**Human Subject Research (involving human participants and/or tissue)**<br>• Give the name of the institutional review board or ethics committee that approved the study<br>• Include the approval number and/or a statement indicating approval of this research<br>• Indicate the form of consent obtained (written/oral) or the reason that consent was not obtained (e.g. the data were analyzed anonymously)<br><br>**Animal Research (involving vertebrate animals, embryos or tissues)**<br>• Provide the name of the Institutional Animal Care and Use Committee (IACUC) or other relevant ethics board that reviewed the study protocol, and indicate whether they approved this research or granted a formal waiver of ethical approval<br>• Include an approval number if one was obtained<br>• If the study involved *non-human primates*, add *additional details* about animal welfare and steps taken to ameliorate suffering<br>• If anesthesia, euthanasia, or any kind of animal sacrifice is part of the study, include briefly which substances and/or methods were applied<br><br>**Field Research**<br><br>Include the following details if this study involves the collection of plant, animal, or other materials from a natural setting:<br>• Field permit number<br>• Name of the institution or relevant body that granted permission	
**Data Availability**<br><br>Authors are required to make all data underlying the findings described fully available, without restriction, and from the time of publication. PLOS allows rare exceptions to address legal and ethical concerns. See the PLOS Data Policy and FAQ for detailed information.	Yes - all data are fully available without restriction

A Data Availability Statement describing where the data can be found is required at submission. Your answers to this question constitute the Data Availability Statement and **will be published in the article**, if accepted.<br><br>**Important:** Stating 'data available on request from the author' is not sufficient. If your data are only available upon request, select 'No' for the first question and explain your exceptional situation in the text box.<br><br>Do the authors confirm that all data underlying the findings described in their manuscript are fully available without restriction?	
**Describe where the data may be found in full sentences. If you are copying our sample text, replace any instances of XXX with the appropriate details.**<br><br>• If the data are **held or will be held in a public repository**, include URLs, accession numbers or DOIs. If this information will only be available after acceptance, indicate this by ticking the box below. For example: *All XXX files are available from the XXX database (accession number(s) XXX, XXX.)*.<br>• If the data are all contained **within the manuscript and/or Supporting Information files**, enter the following: *All relevant data are within the manuscript and its Supporting Information files.*<br>• If neither of these applies but you are able to provide **details of access elsewhere**, with or without limitations, please do so. For example:<br><br>*Data cannot be shared publicly because of [XXX]. Data are available from the XXX Institutional Data Access / Ethics Committee (contact via XXX) for researchers who meet the criteria for access to confidential data.*<br><br>*The data underlying the results presented in the study are available from (include the name of the third party*	All relevant data are within the manuscript and its Supporting Information files.

and contact information or URL).<br>• This text is appropriate if the data are owned by a third party and authors do not have permission to share the data.<br><br>* typeset	
Additional data availability information:	

Powered by Editorial Manager® and ProduXion Manager® from Aries Systems Corporation

Manuscript Click here to access/download;Manuscript;Manuscript.docx

Effects of long-term and high-dose administration of glucocorticoids on the cranial cruciate ligament in healthy beagle dogs

Short title: Glucocorticoid effects on cranial cruciate ligament in dogs

Masakazu Shimada[1]*, Koki Wada[1], Sachiyo Tanaka[1], Sawako Murakami[1], Nobuo Kanno[1], Kei Hayashi[2], Yasushi Hara[1]

Author affiliations:

1) Division of Veterinary Surgery, Department of Veterinary Science, Faculty of Veterinary Medicine, Nippon Veterinary and Life Science University, Musashino-shi, Tokyo, Japan

2) Department of Clinical Sciences, College of Veterinary Medicine, Cornell University, Ithaca, New York, United States

*Corresponding author:masakazu42710@gmail.com

Abstract

This study aimed to determine the effects of long-term and high-dose administration of glucocorticoids (GCs) on the histological and mechanical properties of the cranial cruciate ligament (CrCL) in healthy beagle dogs. A synthetic corticosteroid at 2 mg/kg every 12 h was administered for three months in nine dogs (18 CrCLs) (GC group). Twenty CrCLs from 12 healthy male beagles were used as the normal control (control group). CrCLs were histologically examined (n=12 in the GC group and n=14 in the control group) using hematoxylin-eosin, Alcian-Blue, and Elastica-Eosin stains, and immunohistological staining of type 1 collagen and elastin. An additional 12 CrCLs were mechanically tested (n=6 in the GC group and n=6 in the control groups) to determine failure pattern, maximum tensile strength, maximum stress, elastic modulus, and stress and strain at the transition point. The histological examination revealed a significant increase in interfascicular area and fibrillar disorientation at the tibial attachment in both groups. The ratios of mucopolysaccharide-positive area and positive areas of elastic fibers were significantly higher in the control group than in the GC group. The biomechanical examination demonstrated significantly lower stress at the transition point in the GC group than in the control group. The results of the present study indicate that high-dose corticosteroid may affect metabolism, such as the production of mucopolysaccharides and elastic fibers, although the effect on type 1 collagen production is small. These changes of the extracellular matrix had a small effect on the strength of the ligament. This study suggested that the ligamentous changes associated with GC are different from the degeneration observed in spontaneous canine CrCL disease.

Introduction

Cranial cruciate ligament rupture (CrCLR) is a common cause of hindlimb lameness in dogs. Canine CrCLR is characterized by progressive chronic ligament degeneration with chondrometaplasia, and it is referred to as "cranial cruciate ligament disease" (CCLD) [1, 2]. The ligament is composed of ligamentous cells and extracellular matrix (ECM), of which collagen comprises approximately 75% of the dry weight [3]. Approximately 85% of ligamentous collagens are type 1 collagen (COL1), which attributes to the ligament's resistance to tensile forces [3]. Cranial cruciate ligament (CrCL) degeneration with chondrometaplasia reportedly occurs with increased cartilage matrix, such as type 2 collagen and proteoglycans [2, 4, 5]. The aforementioned degeneration induces the deterioration of mechanical properties and progresses to CrCLR [5]. Despite the unclear pathogenesis of CCLD, researchers have identified numerous associated factors, such as genetics, age, body weight, endocrinological factors, neurological factors, and bone conformation [5-9].

Glucocorticoids (GCs) have various functions, such as an increase in glycogenesis, fat and protein catabolism, and anti-inflammatory effects.GCs suppress fibroblast activity and inhibit ECM synthesis. There have been reports on suspected cases of non-traumatic Achilles tendon rupture in humans, associated with long-term steroid therapy and Cushing's syndrome [10, 11] and an increased risk of patellar tendon rupture, associated with the intra-articular administration of steroids to the knee joint [12]. An association between hypercortisolemia, such as hyperadrenocorticism, and CrCLR in dogs has been reported [13]. To date, no study has yet examined the cause and effect relationship between CrCLR and GC in dogs.

The purpose of this study was to examine the CrCL of healthy beagle dogs that were administered a high dose of the GC. We hypothesized that GCs affect ECM production in CrCLs, thus resulting in ligament fragility. The effects of GCs on the CrCL were investigated by histologically evaluating the degree of CrCL degeneration and by analyzing the tensile biomechanical properties of the CrCL using a universal material testing machine.

Materials & methods

Animals

This study was approved by the Institutional Bioethics Committee of the Experimental Animal Committee (approval number: 2019S-60 and 2020S-25). Table 1 shows the individual information of the 21 male beagles involved in the study. Nine male beagles were orally administered a high dose of a synthetic corticosteroid (prednisolone) at 2 mg/kg every 12 h for 84 days (GC group). This model was created for other unrelated approved studies (approval number: 2019S-72, 29S-37). In the GC group, six dogs (12 stifles) were used for histological analysis and three dogs (six stifles) for biomechanical analysis, respectively. Twenty stifles from 12 healthy male beagles that were euthanized for other unrelated approved studies (approval number: 2019J-29, 30K-9) were used as a normal control group (control group). Of these beagles, fourteen stifles from 7 dogs were used for histological analysis and 6 stifles from 5 dogs were used for biomechanical analysis, respectively. Beagles in both the GC and control groups were housed in cages (0.7 m wide × 1.2 m deep × 1.5 m high), and no other exercise was performed outside the cages. All dogs were euthanized with an overdose of pentobarbital to minimize animal suffering.

Table 1 Individual information

Individual information

	Number of dogs	BW (kg)	Age (months)
Histological analysis			
GC group	6 (12 stifles)	11.1±0.7	15.5±0.5
Control group	7 (14 stifles)	12.1±0.7	20.0±4.0
Biomechanical analysis			

GC group	3 (6 stifles)	11.0±0.9	15.0±0.0
Control group	5 (6 stifles)	8.9±0.5	15.5±4.0

BW: body weight; GC: glucocorticoid.

Data are presented as mean±standard deviation.

Histological and immunohistological analysis

Following euthanasia, the CrCLs were collected and fixed in 4% paraformaldehyde for 24 h. The paraformaldehyde was not changed during the process. Subsequently, a paraffin-embedded procedure was performed and thin longitudinal sections of each specimen were prepared. These specimens were used for hematoxylin-eosin, Alcian-Blue (AB), Elastica-Eosin (EE), and immunohistological staining.

Immunohistological staining was performed for COL1 and elastin. The sections were immersed in methanol containing 3% H_2O_2 for 30 min to avoid endogenous peroxidase activity. Antigen retrieval was performed by incubation in a citrate buffer (0.01 M, pH 6.0) for 60 min at 65°C. While the COL1 sections were blocked with normal rabbit serum, those for elastin were blocked with normal goat serum for 30 min at room temperature before applying the antibodies. The sections were incubated overnight with primary antibodies against COL1 (1:500 dilution; ARG21965 Goat Polyclonal antibody recognized COL1, Arigo Biolaboratories Corp., Hsinchu, Taiwan) and elastin (1:100 dilution; ab21610 Rabbit Polyclonal antibody recognized elastin, Abcam, Cambridge, UK). To confirm the cross-reactivity, appropriate positive controls were included in each immunostaining protocol. The normal canine mammary gland was selected as the positive control for COL1. In contrast, the normal canine aorta was selected as the positive control for elastin. The sections were then incubated with a secondary antibody for 60 min at room temperature and subsequently stained with 3,3′-diaminobenzidine for 2 min. The slides were counterstained with hematoxylin.

The sections were fractionated into three compartments at the tibial, middle, and femoral sides. Three fields of 400x were randomly selected in each compartment and the cell density in the hematoxylin-eosin stained slides was assessed. AB stained slides were used to evaluate the mucopolysaccharide-positive areas and EE was used to evaluate the elastic fiber-positive areas stained with resorcin-fuchsin. For the immunohistological analysis, COL1 positive areas and the elastin positive cells were evaluated. The elastin positive cell density was calculated by dividing the total number of cells in the observed field of view by the area (cells/mm^2). The positive area ratio was calculated by dividing the positive area by the total area x 100. The positive cell ratio was calculated by dividing the number of positive cells by the total number of cells x 100. All data were analyzed using an image analysis software (ImageJ) by a single blinded observer.

Biomechanical analysis

Specimen preparation

Following euthanasia, the tibiofemoral joint was harvested along with the soft tissues surrounding the stifle joint. The specimens were wrapped in lactated Ringer's solution-soaked gauze and stored at -20°C until the test day. All specimens were used within 1 year of the collection. After thawing at 4°C for a day before the test, all soft tissues except the CrCL were removed to create a bone-ligament-bone model. The femur and tibia of each stifle were then sectioned to a length of 10 cm from the joint line. Subsequently, the ends of the femur and tibia were potted in cylindrical molds of acrylic resin (GCOSTRON II I; GC Corporation, Tokyo, Japan). Lactated Ringer's solution was sprayed during the testing to prevent drying after thawing.

Testing protocol

A universal material testing machine (Autograph AGS-X, SHIMADZU Corporation, Kyoto, Japan) was used. The stifle joints were placed at a joint angle of 135° and distracted in

the proximal-distal direction. A preliminary load of 0.5 N was applied to release the creep, followed by a load of 10 N/sec. An approximate straight line of the toe-region was created in the range of 0.05 MPa to 0.2 MPa, which showed linear behavior in all specimens following preliminary loading. The transition point was determined, which was the intersection of the approximate straight line of the toe-region and the approximate straight line of the liner-region (Fig 1) [14]. The following parameters were recorded and analyzed: the failure pattern, maximum tensile strength (N), maximum stress (MPa) [maximum tensile strength (N) / sectional area (mm^2)], elastic modulus [stress (N) / strain (%)], stress at the transition point (MPa), and strain at the transition point (%). The CrCLs were transected perpendicular to the longitudinal axis of ligament with a scalpel at its attachments to femur and tibia, and the section was stained with India ink to measure the femoral and tibial cross-sectional areas using Image J (Fig 2). The average of the two cross-sectional areas from both sides was used as the cross-sectional area of the CrCL.

Fig 1. The stress-strain curve. The transition point (yellow dot) has been set at the intersection of the toe-region approximation line and the liner-region approximation line following the creation of the stress-displacement curve.

Fig 2. Photograph used to measure the area of the femoral side in Image J. The cranial cruciate ligament was transected perpendicular to the longitudinal axis of ligament with a scalpel at its attachments to the femur and tibia, and the section was stained with India ink to measure the cross-sectional area using Image J. To calibrate the length of the object, the photograph was taken at the same time the scale was taken.

Statistics analyses

SPSS software version 26 (SPSS Inc., Chicago, IL, USA) was used for all statistical analyses. Statistical analysis included comparison of the differences in the areas of each group

from the histological and immunohistological analyses. The Kruskal Wallis test was followed by the Mann-Whitney test as a post-hoc test. In addition, the Mann-Whitney test was performed to compare the differences among the histological, immunohistological, and biomechanical analyses within each group. Differences were considered statistically significant at a P-value <0.05.

Results

Histological and immunohistological analysis

There was no macroscopic abnormality in the CrCL in either group at the time of euthanasia.

The interfascicular area and fibrocartilage were more extensive at the ligament attachments, particularly on the tibial side, than in the middle within each group (Fig 3). In addition, irregularities in ligamentous fibril orientation were observed on the tibial side. There were no significant differences in the cell density between the areas within each group and between the groups.

Fig 3. Hematoxylin-eosin staining findings of the cranial cruciate ligament in the GC and control groups (bar=20 μm). The upper panels represent examples of staining on the tibial (A), middle (B), and femoral (C) sides of the GC group. The lower panels represent examples of staining on the tibial (D), middle (E), and femoral (F) sides of the control group. In the box-and-whisker plot, blue, orange, gray, and yellow indicate the tibial side, middle, femoral side, and the entire cell count, respectively.

Within each group, the interfascicular area and fibrocartilage are more extensive at the ligament attachments, particularly on the tibial side, than in the middle. Irregularities are observed in fibril orientation. In the analysis of cell density, there are no significant differences

between the areas within each group and between the groups.

GC, glucocorticoid.

Areas with positive AB staining were particularly obvious in the areas of irregular fibril orientation around the round cells, such as in the interfascicular area and fibrocartilage (Fig 4). Within each group, positive AB staining area ratio was significantly higher on the tibial side than on the femoral side and in the middle. The ratio was significantly higher in the control group than in the GC group on the tibial side.

Fig 4. Alcian-blue staining findings of the cranial cruciate ligament in the GC and control groups (bar=20 µm). The upper panels represent examples of staining on the tibial (A), middle (B), and femoral (C) sides of the GC group. The lower panels represent examples of staining on the tibial (D), middle (E), and femoral (F) sides of the control group. In the box-and-whisker plot, blue, orange, gray, and yellow indicate the tibial side, middle, femoral side, and the entire measurement, respectively. The asterisks indicate $P<0.05$.

Areas with positive Alcian-blue staining are particularly obvious in the areas of irregular fibril orientation around the round cells, such as the interfascicular area and fibrocartilage. Within each group, positive Alcian-blue area ratio is significantly higher on the tibial side than on the femoral side and in the middle. The ratio is significantly higher in the control group than in the GC group on the tibial side.

GC, glucocorticoid.

Areas with positive EE staining were observed, particularly in the interfascicular area and fibrocartilage (Fig 5). Positive EE area ratio was significantly higher on the tibial side than on the femoral side and in the middle for the control group. The ratio was significantly higher at the tibial side, middle, femoral side, and entire areas in the control group compared to the GC group.

Fig 5. Elastica-Eosin staining findings of the cranial cruciate ligament in the GC and control groups (bar=20 μm). The upper panels represent examples of staining on the tibial (A), middle (B), and femoral (C) sides of the GC group. The lower panels represent examples of staining on the tibial (D), middle (E), and femoral (F) sides of the control group. In the box-and-whisker plot, blue, orange, gray, and yellow indicate the tibial side, middle, femoral side, and the entire measurement, respectively. The asterisks indicate $P<0.05$.
Positive areas of Elastica-Eosin are observed, particularly in the interfascicular area and fibrocartilage. In the control group, the ratio is significantly higher on the tibial side than on the femoral side and in the middle. The ratio is significantly higher in the control group compared to the GC group in the tibial, middle, femoral, and entire areas.
GC, glucocorticoid.

Extensive positive areas of COL1 immunohistochemical staining were found in areas with normal ligament fibril orientation (Figs 6 and 7). While analyzing the positive ratio, there were no significant differences between the areas within each group and between the groups.

Fig 6. Immunohistochemical staining of type 1 collagen findings of the cranial cruciate ligament in the GC and control groups (bar=20 μm). The upper panels depict examples of staining on the tibial (A), middle (B), and femoral (C) sides of the GC group. The lower panels depict examples of staining on the tibial (D), middle (E), and femoral (F) sides of the control group. In the box-and-whisker plot, blue, orange, gray, and yellow indicate the tibial side, middle, femoral side, and the entire measurement, respectively.
Extensive positive regions are found in areas with normal ligament orientation. In the analysis of the positive ratio, there are no significant differences between the areas within each group and between the groups.
GC, glucocorticoid.

Fig 7. Positive control of immunohistochemical staining. A: Using the mammary gland as a positive control for type 1 collagen, a positive area is found in the dermis. B: A positive area is found in the intracellular area of the mesothelial area using the normal canine aorta, selected as the positive control for elastin.

The area ratio of positive elastin immunohistochemical staining was significantly higher on the tibial side than on the femoral side and the middle in the control group (Fig 8). The ratio was significantly higher in the control group than in the GC group on the tibial side.

Fig 8. Immunohistochemical staining of the elastin findings of the cranial cruciate ligament in the GC and control groups (bar=20 μm). The upper panels depict examples of staining on the tibial (A), middle (B), and femoral (C) sides of the GC group. The lower panels depict examples of staining on the tibial (D), middle (E), and femoral (F) sides of the control group. In the box-and-whisker plot, blue, orange, gray, and yellow indicate the tibial side, middle, femoral side, and the entire measurement, respectively. The asterisks indicate $P<0.05$. In the control group, the ratio is significantly higher on the tibial side than on the femoral side and in the middle. The ratio is significantly higher in the control group than in the GC group on the tibial side.

GC, glucocorticoid.

Biomechanical analysis

The failure pattern in both groups demonstrated an avulsion fracture at the tibial attachment in all specimens (Fig 9). One specimen from the control group had an avulsion fracture of the tibia with a partial ligament tear. The maximum tensile strength, maximum stress, elastic modulus, and strain at the transition point did not differ significantly between the groups. The stress at the transition point was significantly lower in the GC group than in the control

group (Fig 10).

Fig 9. Failure pattern of the biomechanical test. A: The femoral side after the failure. The end of the ligament is attached to the bony fragment of the tibia (red circle). B: The tibial side after the failure. The cranial cruciate ligament is detached over the entire area (red circle).

Fig 10. Tensile test results of the bone-ligament-bone model in the GC and control groups. A: stress at the transition point; B: strain at the transition point; C: elastic modulus; D: maximum tensile strength; and E: maximum stress. The asterisks indicate P<0.05. The stress at the transition point is significantly lower in the GC group than in the control group.
GC, glucocorticoid.

Discussion

No previous study has examined the effects of high-dose administration of GC on the CrCL in dogs. The results of the present study indicate that a high-dose of prednisolone may affect metabolism, such as the production of mucopolysaccharides and elastic fibers, although the effect on COL1 production, the major ECM, is small. The change of ECM had a small effect on the strength of the ligament, but it was shown to have an effect in the toe region. In conlusion, the ligamentous changes associated with GC were found to be different from the typical degeneration observed in spontaneous CCLD.

CCLD is characterized by a decrease in COL1 along with chondrometaplasia, in addition to an increase in mucopolysaccharides, a representative of the cartilage matrix [2, 4, 5]. In addition, recent reports have shown that elastic fibers increase with ligament degeneration in Labrador Retrievers [15]. Elastic fibers are considered a minor ECM in ligaments [3]. However, Smith et al. reported that they account for 9.86±3.97% of the dry weight in greyhound CrCLs [16]. Interestingly, greyhounds, a breed known to have a low incidence of CrCLR, have been reported to have more elastic fibers in their CrCL than those in Labrador Retrievers [15, 16]. Therefore, it

is possible that greyhounds compensate for CrCL damage by producing elastic fibers. Elastic fibers may be associated with CrCL repair and the difference in the balance between repair and damage may be reflected in the risk of CrCLR [15]. Despite the unclear pathogenesis of CCLD, researchers have identified numerous associated factors. In humans, non-traumatic Achilles tendon rupture has been reported to be associated with Cushing's syndrome and long-term steroid therapy [10, 11]. Similarly, hypercorticoidemia in horses is a risk factor for injury [17, 18]. These studies reported collagen misalignment and mucopolysaccharide deposition, similar to canine CCLD, as features of suspensory ligament degeneration in horses with pituitary pars intermedia dysfunction [17, 18].

In both the GC and control groups, the increased production of mucopolysaccharides were observed in areas where there were irregularities in ligament orientation in the ligamentous attachments of the tibia. There were more elastin-positive cells and elastic fibers in the tibial attachments than in other regions in the control group. More elastic fibers were produced in areas with pronounced irregular fibril orientation in the control group, such as in the area of tibial attachments. In contrast, fewer elastic fibers were produced throughout the ligament in the GC group, compared to the control group. In this study, CCLD-like degeneration was expected to occur as in the equine suspensory ligament, but no deposition of mucopolysaccharides associated with hypercorticoidemia was observed. In vitro studies have reported that the administration of GC preparations to tenocytes inhibits the production of collagen and proteoglycans [19, 20]. The production of ECM components was possibly suppressed in this study, as well as in these effects, but the effect on COL1 was minimal. The glucocorticoid receptor is down-regulated with long-term GC administration in dogs and humans [21, 22]. However, the number of glucocorticoid receptor-positive cells rather increased in the suspensory ligament of horses with pituitary pars intermedia dysfunction [18]. Differences in the changes associated with hypercortisolemia in horses may be attributed to the differences in exposure to blood GC concentrations between the endocrine disease with chronic hypercorticoidemia and the experimental model with high-dose GC preparations and the exercise restriction-induced

limitation of chronic mechanical loading on the ligaments. This might have resulted in a different mechanism for CCLD-like degeneration. Thus, various factors are intricately involved in the bioactivity of GCs and their regulation. Moreover, it is still unclear if the abovementioned events occur in all tissues in a similar way.

The failure of all specimens in the biomechanical analysis is attributed to the detachment at the tibial CrCL attachment as in a previous study using tensile tests [23]. This may be a consequence of changes in the ECM component at the tibial attachment where the transition occurs between ligament and cartilage with changes in material properties, thus causing stress concentration [24, 25]. Therefore, in young healthy beagle dogs with minimal ligament weakening associated with CCLD, stress was thought to be concentrated at the ligament origin, and detachment occurred. A significant difference in stress at the transition point was observed, similar to the results reported using the porcine medial collateral ligament [26]. In other words, the transition between the toe region and liner region was smooth, owing to the presence of elastic fibers in the control group. Considering the absence of elastic fibers in the GC group, it was not possible to provide sufficient pre-stress to the collagen fibers, suggesting the risk of micro-damage to the ligament. Thus, our results suggest that CrCL changes associated with high-dose GC administration may be different from that of canine CCLD, which in turn is characterized by the chondrogenesis of the CrCL.

Our study had several limitations. First, different beagle dogs were used for the histological and biomechanical studies. The relationship between the histological and biomechanical analysis could have been directly examined using one side for each analysis, however, beagle dogs created for other purposes were used in this study. Thus, the histological study was initially conducted, which was followed at a later stage by the biomechanical study. Second, it was difficult to set up the ligament on the universal material testing machine for the biomechanical study. Thus, the femur and tibia needed to be clamped for the experiment. Moreover, it was not possible to completely investigate the material properties of the ligament because of the bone detachment that occurred at the CrCL attachment. Third, the cross-sectional

area of the ligament was calculated after the experiment. Thus, it may differ from the original cross-sectional area because of elongation. Fourth, higher doses and longer durations of GCs were administered than those used in clinical practice to clarify the effects of GCs. Therefore, our results do not necessarily reflect Cushing's syndrome in clinical cases or the degeneration of the CrCL in the case of long-term GC administration.

Our findings suggested that high-dose GC treatment for three months in healthy dogs suppressed the production of mucopolysaccharides and elastic fibers, although the effect on COL1 production was small. The change of ECM had a small effect on the strength of the ligament, but it was shown to affect the toe region. In conclusion, the ligamentous changes associated with GC were found to be different from the degeneration observed in spontaneous CCLD. Even though the role of elastic fibers is still uncertain, it is unclear to what extent prolonged exposure to hypercorticoidemia is a risk factor for CrCLR in dogs. Future epidemiological studies are expected to clarify the relationship between CrCLR and hypercorticoidemia.

Acknowledgments

We gratefully acknowledge the work of the past and present members of our laboratory. We also thank Editage (www.editage.com) for their English language editing service.

References

1. Hayashi K, Manley PA, Muir P. Cranial cruciate ligament pathophysiology in dogs with cruciate disease: a review. J Am Anim Hosp Assoc. 2004;40(5):385-90. Epub 2004/09/07. doi: 10.5326/0400385. PubMed PMID: 15347618.
2. Ichinohe T, Kanno N, Harada Y, Yogo T, Tagawa M, Soeta S, et al. Degenerative changes of the cranial cruciate ligament harvested from dogs with cranial cruciate ligament rupture. J Vet Med Sci. 2015;77(7):761-70. Epub 2015/02/27. doi: 10.1292/jvms.14-0383. PubMed PMID: 25716871; PubMed Central PMCID: PMCPMC4527496.
3. Frank CB. Ligament structure, physiology and function. J Musculoskelet Neuronal Interact. 2004;4(2):199-201. Epub 2004/12/24. PubMed PMID: 15615126.
4. Kyllar M, Cizek P. Cranial cruciate ligament structure in relation to the tibial plateau slope and intercondylar notch width in dogs. J Vet Sci. 2018;19(5):699-707. Epub 2018/06/23. doi: 10.4142/jvs.2018.19.5.699. PubMed PMID: 29929359; PubMed Central PMCID: PMCPMC6167346.
5. Vasseur PB, Pool RR, Arnoczky SP, Lau RE. Correlative biomechanical and histologic study of the cranial cruciate ligament in dogs. Am J Vet Res. 1985;46(9):1842-54. Epub 1985/09/01. PubMed PMID: 3901837.
6. Comerford EJ, Smith K, Hayashi K. Update on the aetiopathogenesis of canine cranial cruciate ligament disease. Vet Comp Orthop Traumatol. 2011;24(2):91-8. Epub 2011/01/19. doi: 10.3415/VCOT-10-04-0055. PubMed PMID: 21243176.
7. Ichinohe T, Kanno N, Harada Y, Yogo T, Tagawa M, Hara Y. Histological and immunohistological analysis of degenerative changes in the cranial cruciate ligament in a canine model of excessive tibial plateau angle. Vet Comp Orthop Traumatol. 2015;28(4):240-9. Epub 2015/05/23. doi: 10.3415/VCOT-14-08-0128. PubMed PMID: 25998226.
8. Hayes GM, Granger N, Langley-Hobbs SJ, Jeffery ND. Abnormal reflex activation of hamstring muscles in dogs with cranial cruciate ligament rupture. Vet J. 2013;196(3):345-50. Epub 2012/12/12. doi: 10.1016/j.tvjl.2012.10.028. PubMed PMID: 23219226.
9. Cook JL. Cranial cruciate ligament disease in dogs: biology versus biomechanics. Vet Surg. 2010;39(3):270-7. Epub 2010/06/05. doi: 10.1111/j.1532-950X.2010.00653.x. PubMed PMID: 20522208.
10. Haines JF. Bilateral rupture of the Achilles tendon in patients on steroid therapy. Ann Rheum Dis. 1983;42(6):652-4. Epub 1983/12/01. doi: 10.1136/ard.42.6.652. PubMed PMID: 6651370; PubMed Central PMCID: PMCPMC1001322.
11. Mousa A, Jones S, Toft A, Perros P. Spontaneous rupture of Achilles tendon: missed presentation of Cushing's syndrome. BMJ. 1999;319(7209):560-1. Epub 1999/08/27. doi:

10.1136/bmj.319.7209.560. PubMed PMID: 10463901; PubMed Central PMCID: PMCPMC1116439.

12. Chen SK, Lu CC, Chou PH, Guo LY, Wu WL. Patellar tendon ruptures in weight lifters after local steroid injections. Arch Orthop Trauma Surg. 2009;129(3):369-72. Epub 2008/06/26. doi: 10.1007/s00402-008-0655-1. PubMed PMID: 18575877.

13. Feldman EC, Nelson RW. Canine hyperadrenocorticism (Cushing's syndrome). In: Canine and Feline Endocrinology and Reproduction. 3rd ed. Missouri: Saunders; 2004. pp. 252-357.

14. Lake SP, Miller KS, Elliott DM, Soslowsky LJ. Tensile properties and fiber alignment of human supraspinatus tendon in the transverse direction demonstrate inhomogeneity, nonlinearity, and regional isotropy. J Biomech. 2010;43(4):727-32. Epub 2009/11/11. doi: 10.1016/j.jbiomech.2009.10.017. PubMed PMID: 19900677; PubMed Central PMCID: PMCPMC2823853.

15. Smith KD, Hayashi K, Clements DN, Clegg PD, Innes JF, Comerford EJ. Variation in the Quantity of Elastic Fibres with Degeneration in Canine Cranial Cruciate Ligaments from Labrador Retrievers. Vet Comp Orthop Traumatol. 2017;30(6):398-402. Epub 2017/12/05. doi: 10.3415/VCOT-17-02-0019. PubMed PMID: 29202502.

16. Smith KD, Clegg PD, Innes JF, Comerford EJ. Elastin content is high in the canine cruciate ligament and is associated with degeneration. Vet J. 2014;199(1):169-74. Epub 2013/12/10. doi: 10.1016/j.tvjl.2013.11.002. PubMed PMID: 24314717; PubMed Central PMCID: PMCPMC6419147.

17. Hofberger S, Gauff F, Licka T. Suspensory ligament degeneration associated with pituitary pars intermedia dysfunction in horses. Vet J. 2015;203(3):348-50. Epub 2015/02/03. doi: 10.1016/j.tvjl.2014.12.037. PubMed PMID: 25641552.

18. Hofberger SC, Gauff F, Thaller D, Morgan R, Keen JA, Licka TF. Assessment of tissue-specific cortisol activity with regard to degeneration of the suspensory ligaments in horses with pituitary pars intermedia dysfunction. Am J Vet Res. 2018;79(2):199-210. Epub 2018/01/24. doi: 10.2460/ajvr.79.2.199. PubMed PMID: 29359976.

19. Wong MW, Tang YY, Lee SK, Fu BS. Glucocorticoids suppress proteoglycan production by human tenocytes. Acta Orthop. 2005;76(6):927-31. Epub 2006/02/14. doi: 10.1080/17453670610046118. PubMed PMID: 16470453.

20. Spang C, Chen J, Backman LJ. The tenocyte phenotype of human primary tendon cells in vitro is reduced by glucocorticoids. BMC Musculoskelet Disord. 2016;17(1):467. Epub 2016/11/12. doi: 10.1186/s12891-016-1328-9. PubMed PMID: 27832770; PubMed Central PMCID: PMCPMC5105245.

21. Gross KL, Lu NZ, Cidlowski JA. Molecular mechanisms regulating glucocorticoid sensitivity and resistance. Mol Cell Endocrinol. 2009;300(1-2):7-16. Epub 2008/11/13. doi:

10.1016/j.mce.2008.10.001. PubMed PMID: 19000736; PubMed Central PMCID: PMCPMC2674248.

22. Tanaka S, Shibuya H, Suzuki S, Kanno N, Harada Y, Sato A, et al. Long-term administration of prednisolone: Effects on the myocardial tissue of healthy beagle dogs. J Vet Med Sci. 2021;83(1):84-93. Epub 2020/12/04. doi: 10.1292/jvms.20-0401. PubMed PMID: 33268642; PubMed Central PMCID: PMCPMC7870400.

23. Wingfield C, Amis AA, Stead AC, Law HT. Comparison of the biomechanical properties of rottweiler and racing greyhound cranial cruciate ligaments. J Small Anim Pract. 2000;41(7):303-7. Epub 2000/09/08. doi: 10.1111/j.1748-5827.2000.tb03206.x. PubMed PMID: 10976625.

24. Benjamin M, Toumi H, Ralphs JR, Bydder G, Best TM, Milz S. Where tendons and ligaments meet bone: attachment sites ('entheses') in relation to exercise and/or mechanical load. J Anat. 2006;208(4):471-90. Epub 2006/04/28. doi: 10.1111/j.1469-7580.2006.00540.x. PubMed PMID: 16637873; PubMed Central PMCID: PMCPMC2100202.

25. Petersen W, Tillmann B. Structure and vascularization of the cruciate ligaments of the human knee joint. Anat Embryol (Berl). 1999;200(3):325-34. Epub 1999/08/27. doi: 10.1007/s004290050283. PubMed PMID: 10463347.

26. Henninger HB, Underwood CJ, Romney SJ, Davis GL, Weiss JA. Effect of elastin digestion on the quasi-static tensile response of medial collateral ligament. J Orthop Res. 2013;31(8):1226-33. Epub 2013/04/05. doi: 10.1002/jor.22352. PubMed PMID: 23553827; PubMed Central PMCID: PMCPMC4112956.

Figure 1

Click here to access/download;Figure;Figure 1.tif

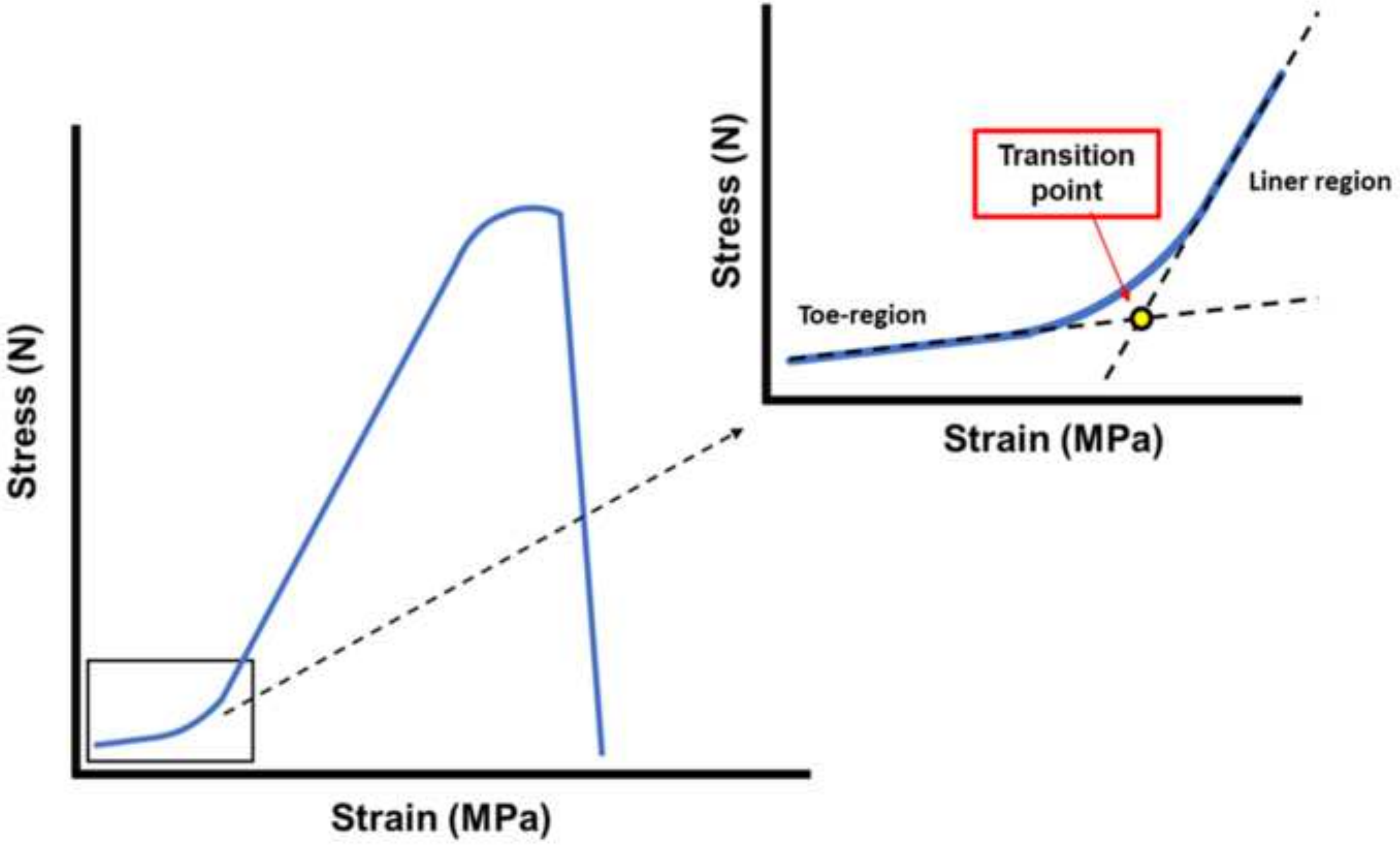

Figure 2

Click here to access/download;Figure;Figure 2 .tif

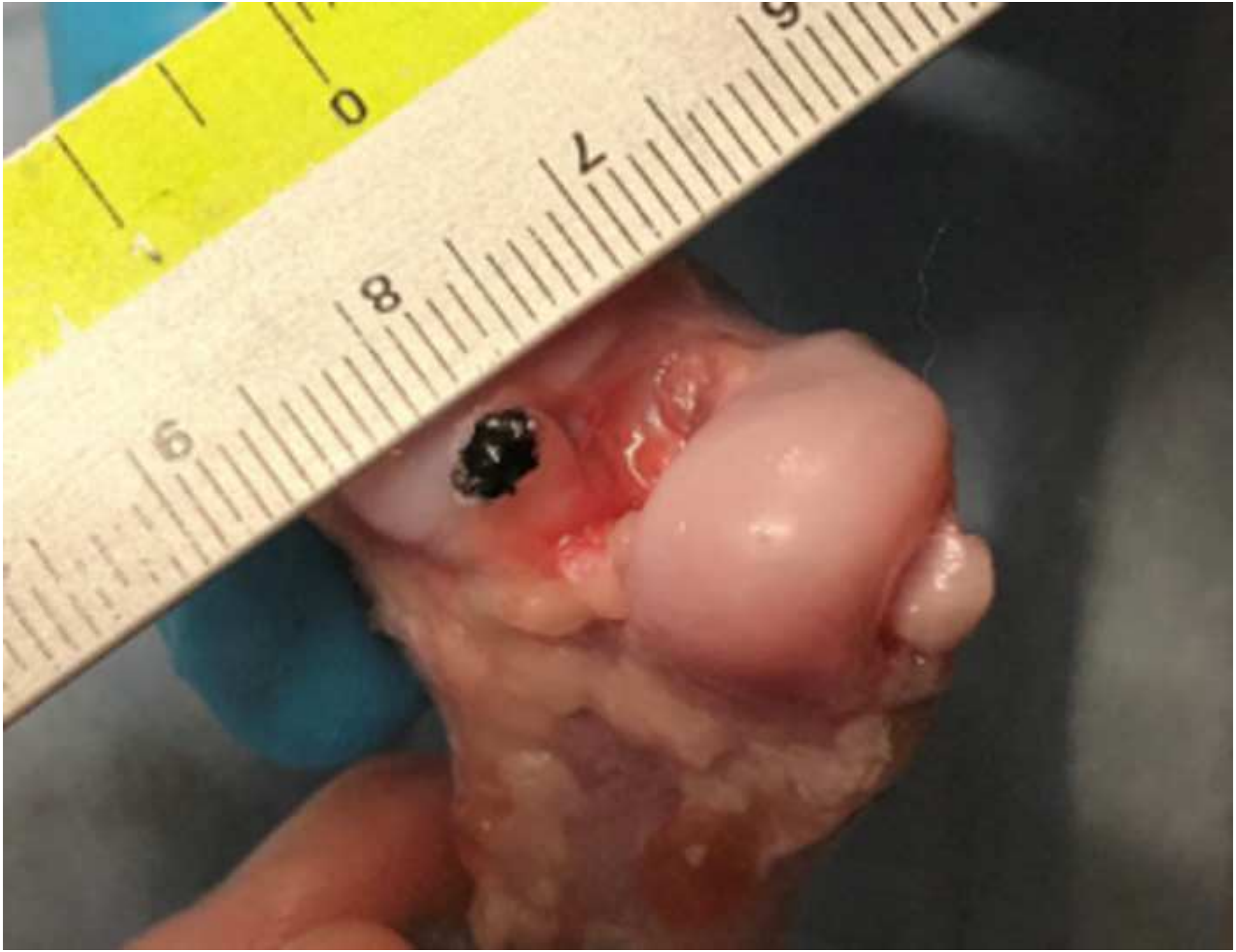

Figure 3

Click here to access/download;Figure;Figure 3.tif

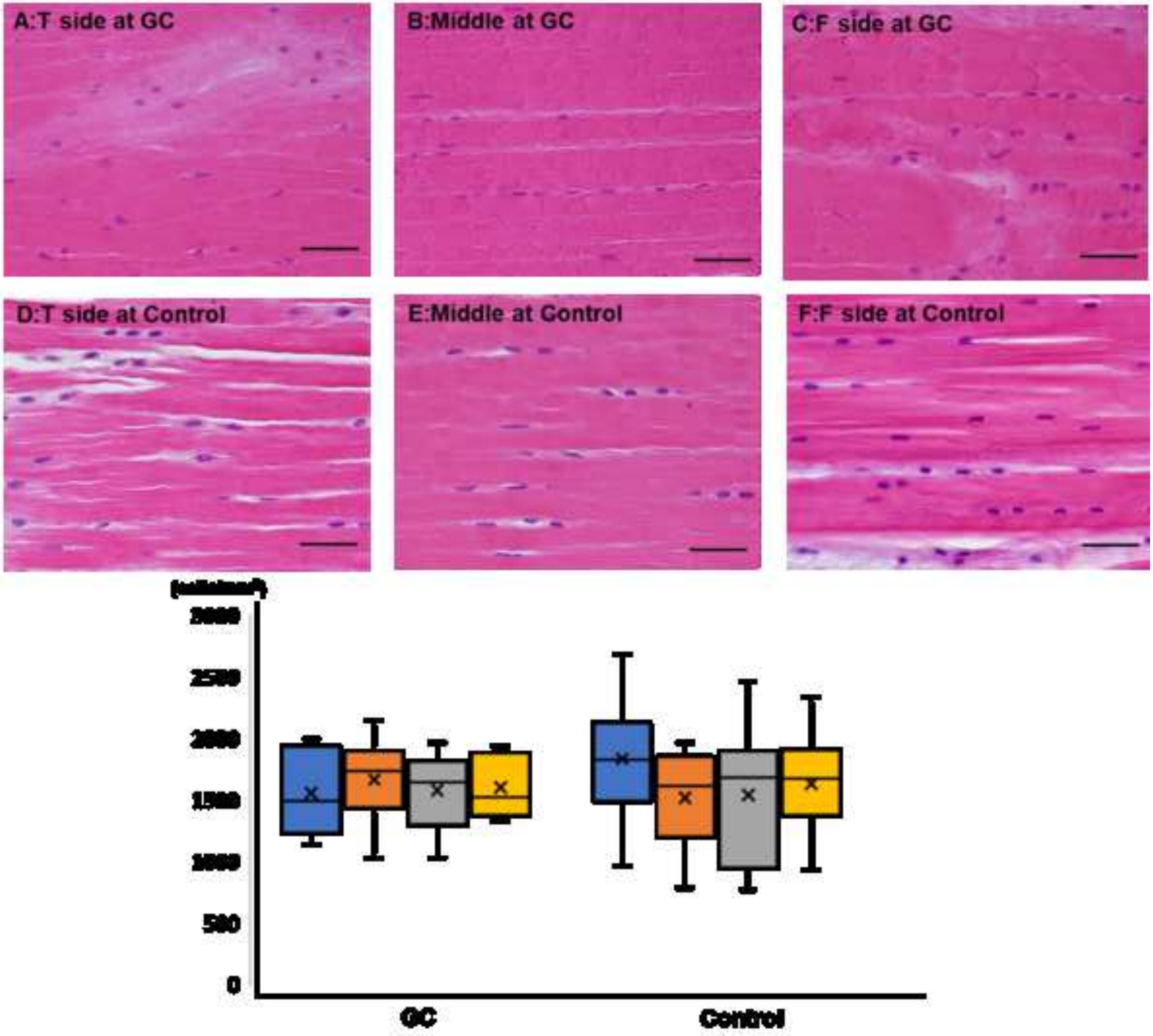

Figure 4 Click here to access/download;Figure;Figure 4.tif

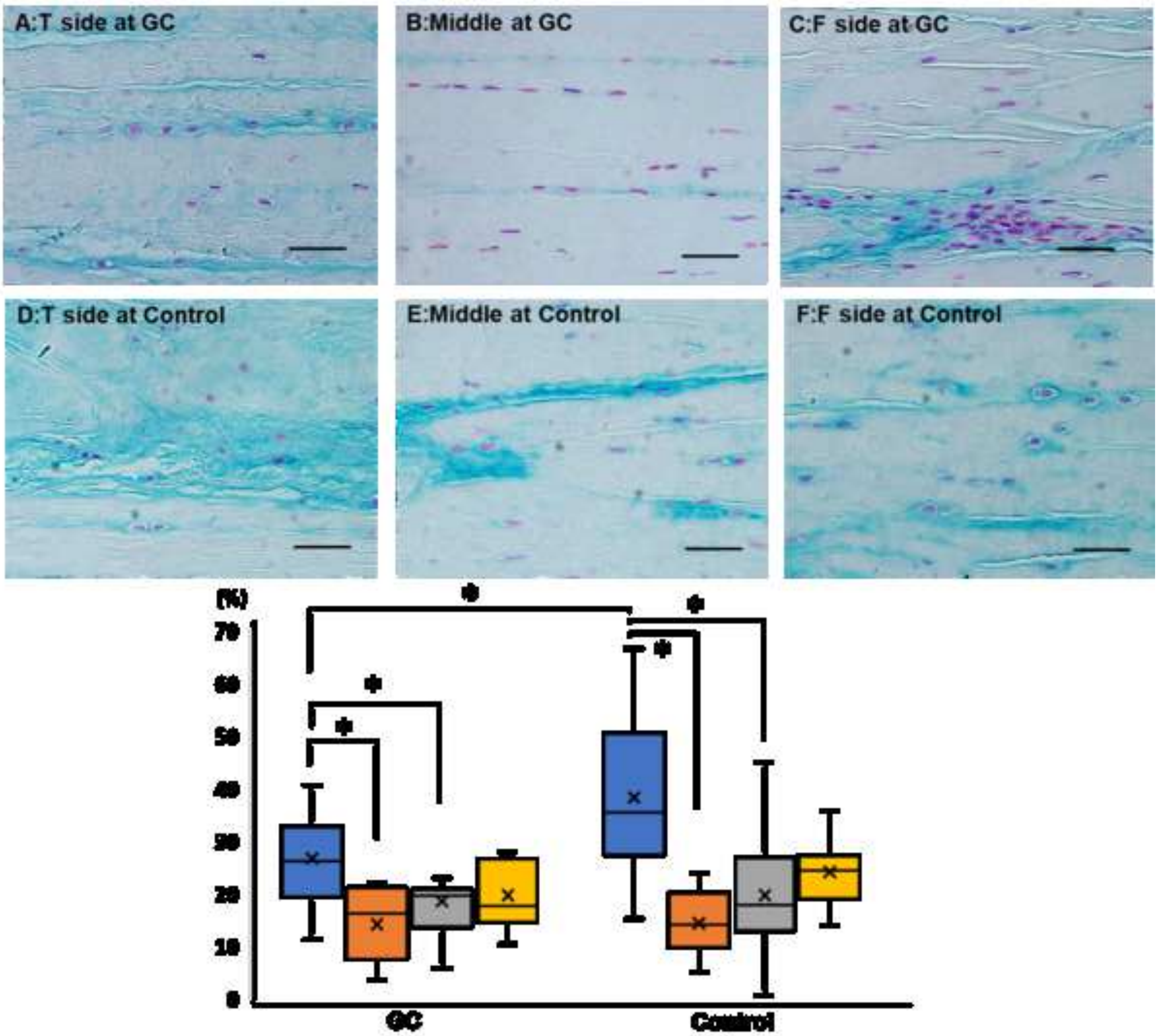

Figure 5

Click here to access/download;Figure;Figure 5.tif

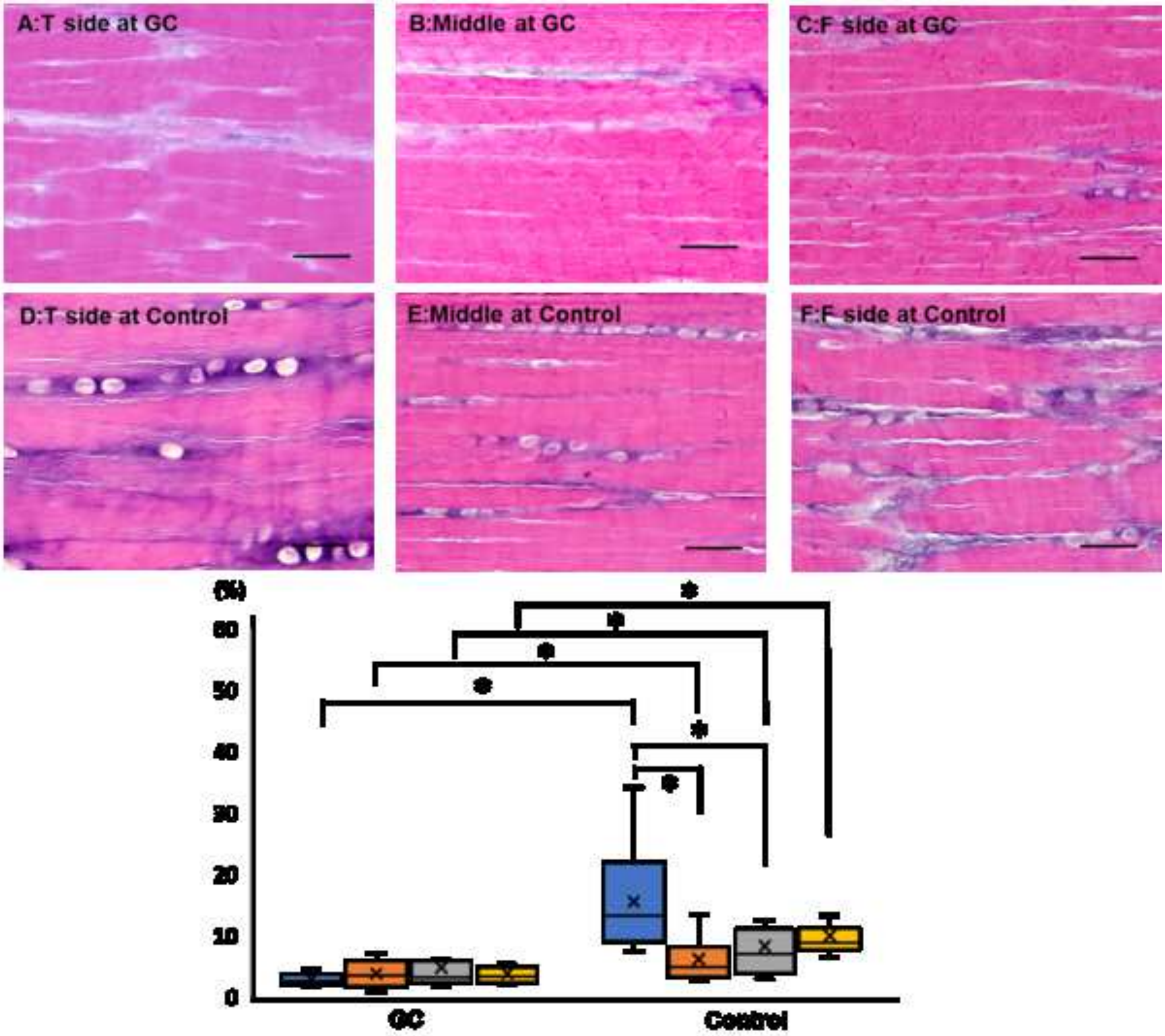

Figure 6

Click here to access/download;Figure;Figure 6.tif

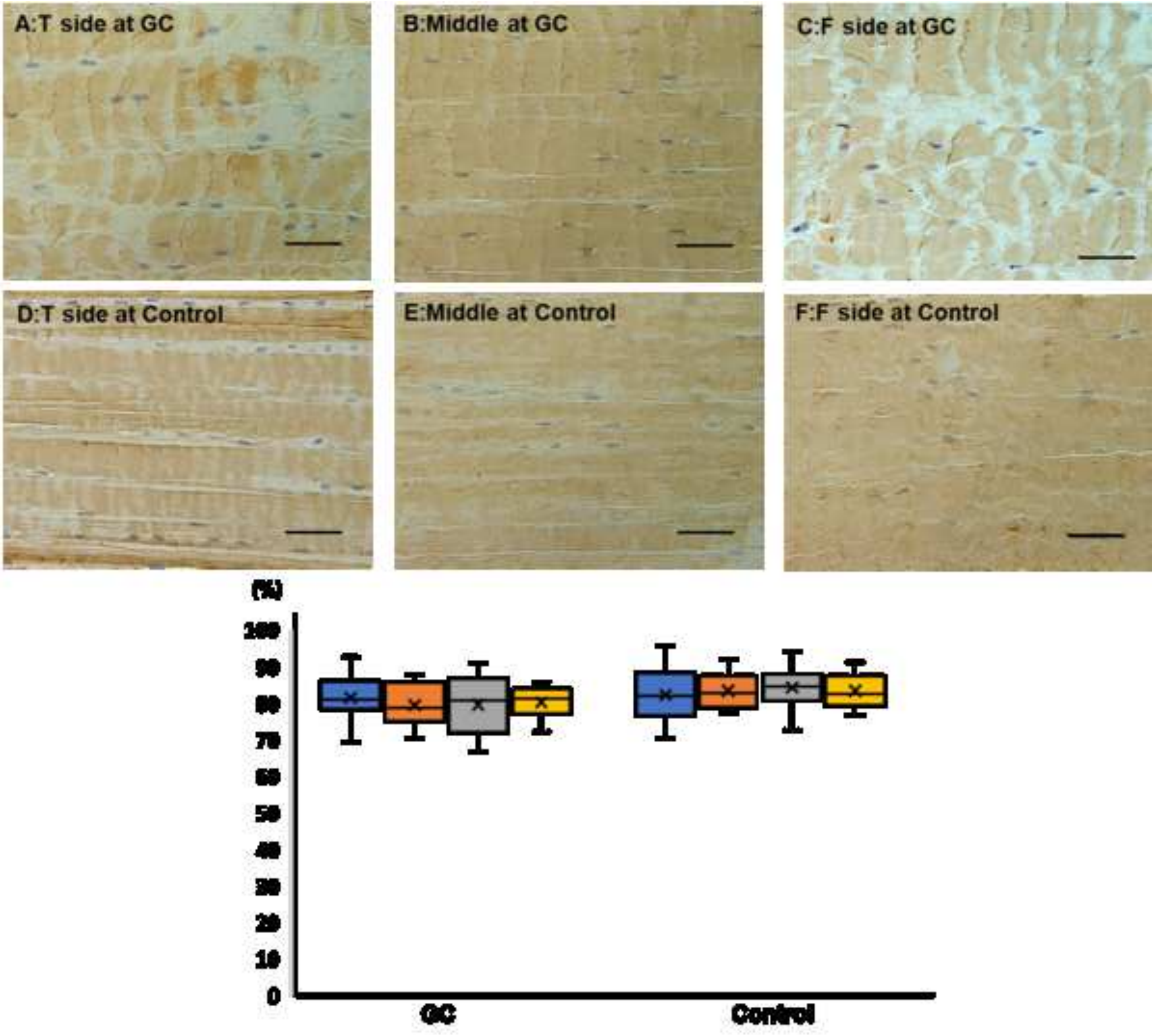

 Click here to access/download;Figure;Figure 7.tif

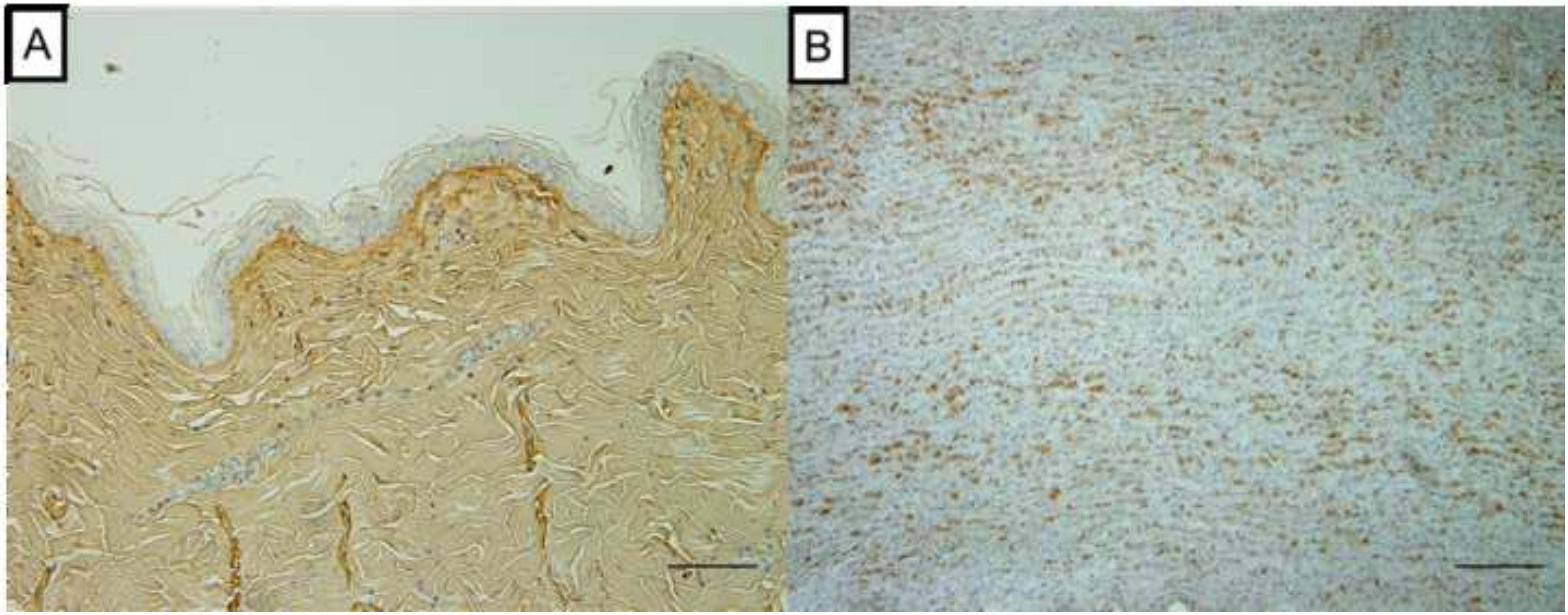

Figure 8

Click here to access/download;Figure;Figure 8.tif

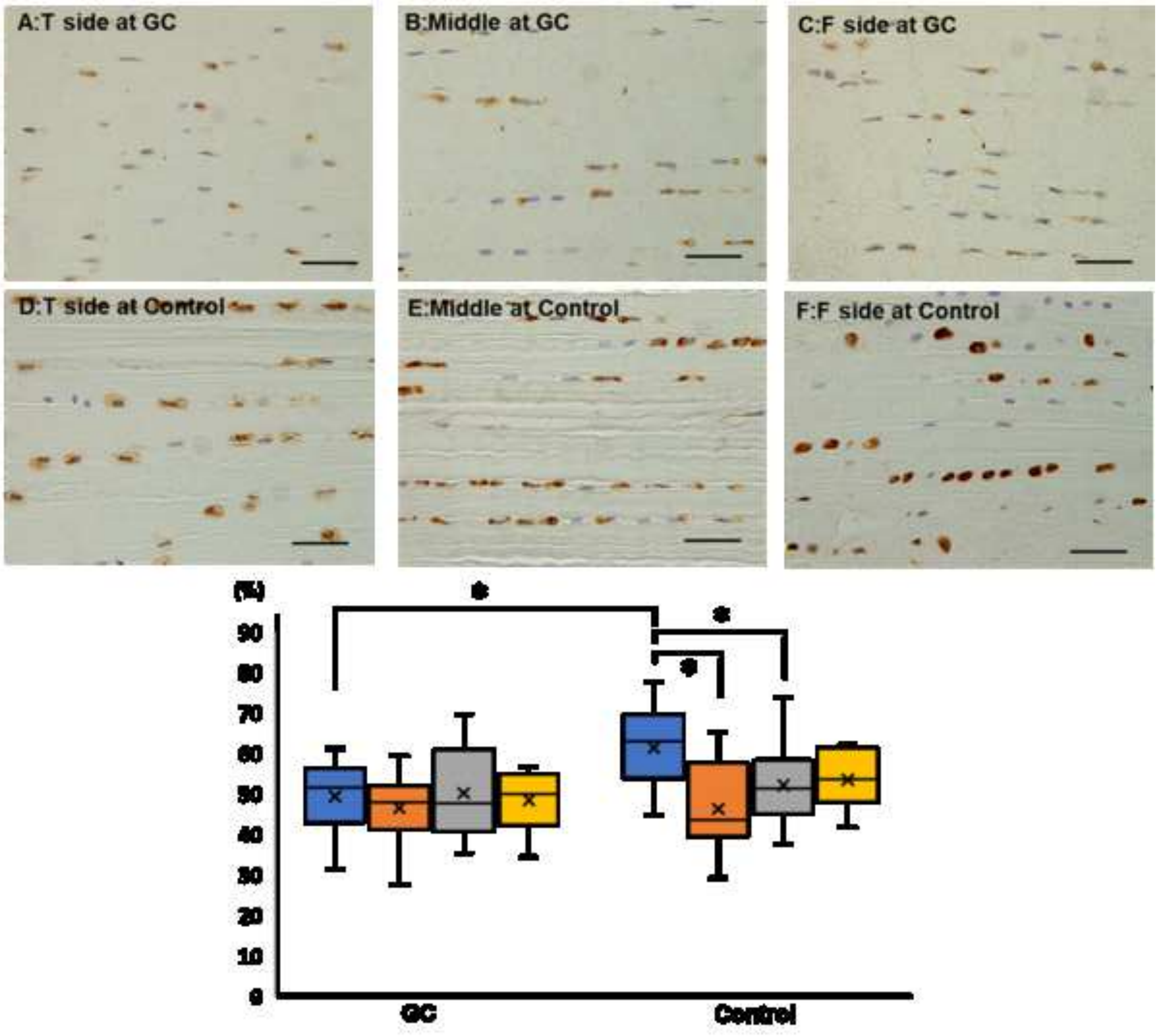

Figure 9

Click here to access/download;Figure;Figure 9.tif

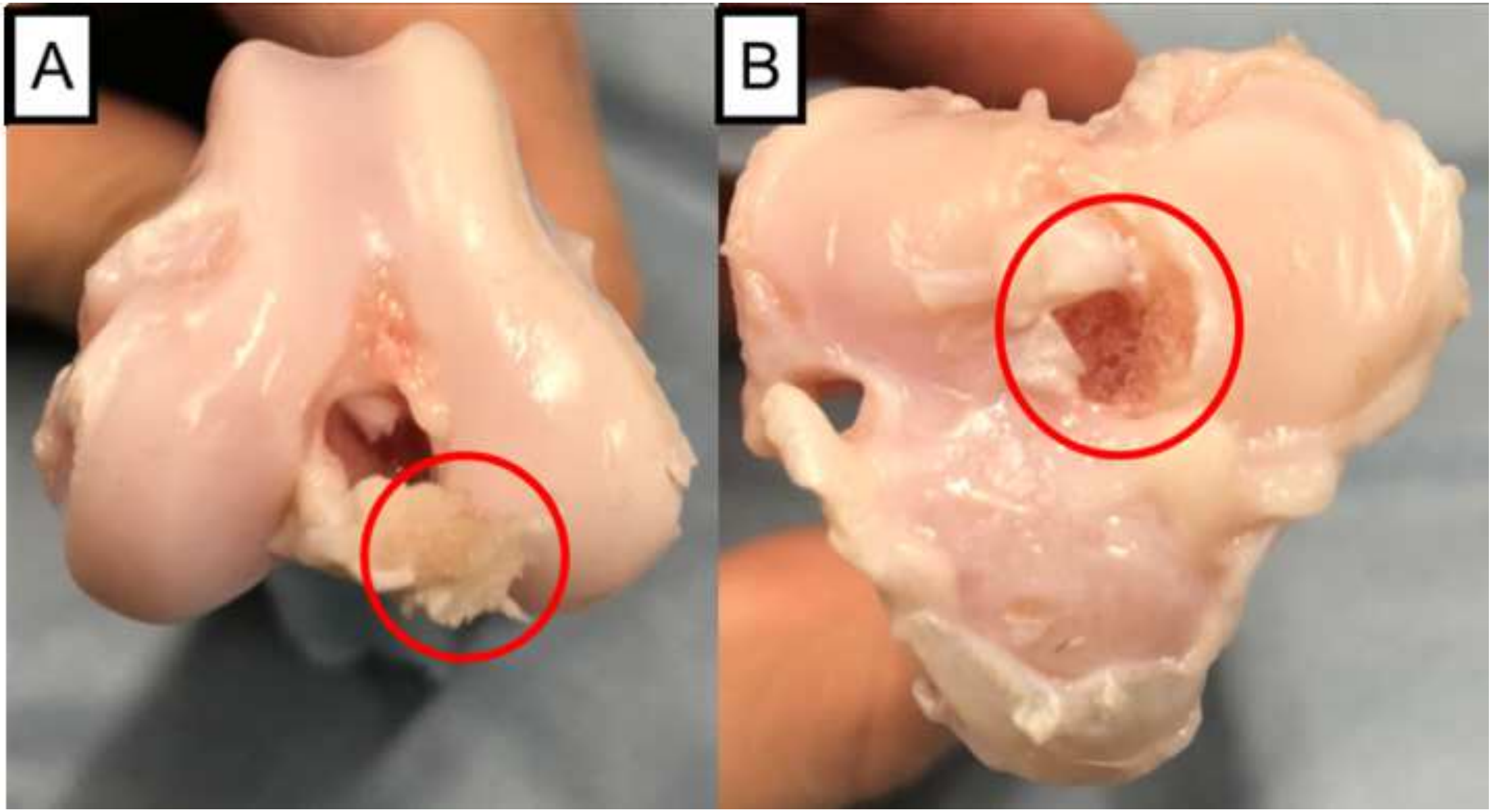

Figure 10

Click here to access/download;Figure;Figure 10.tif

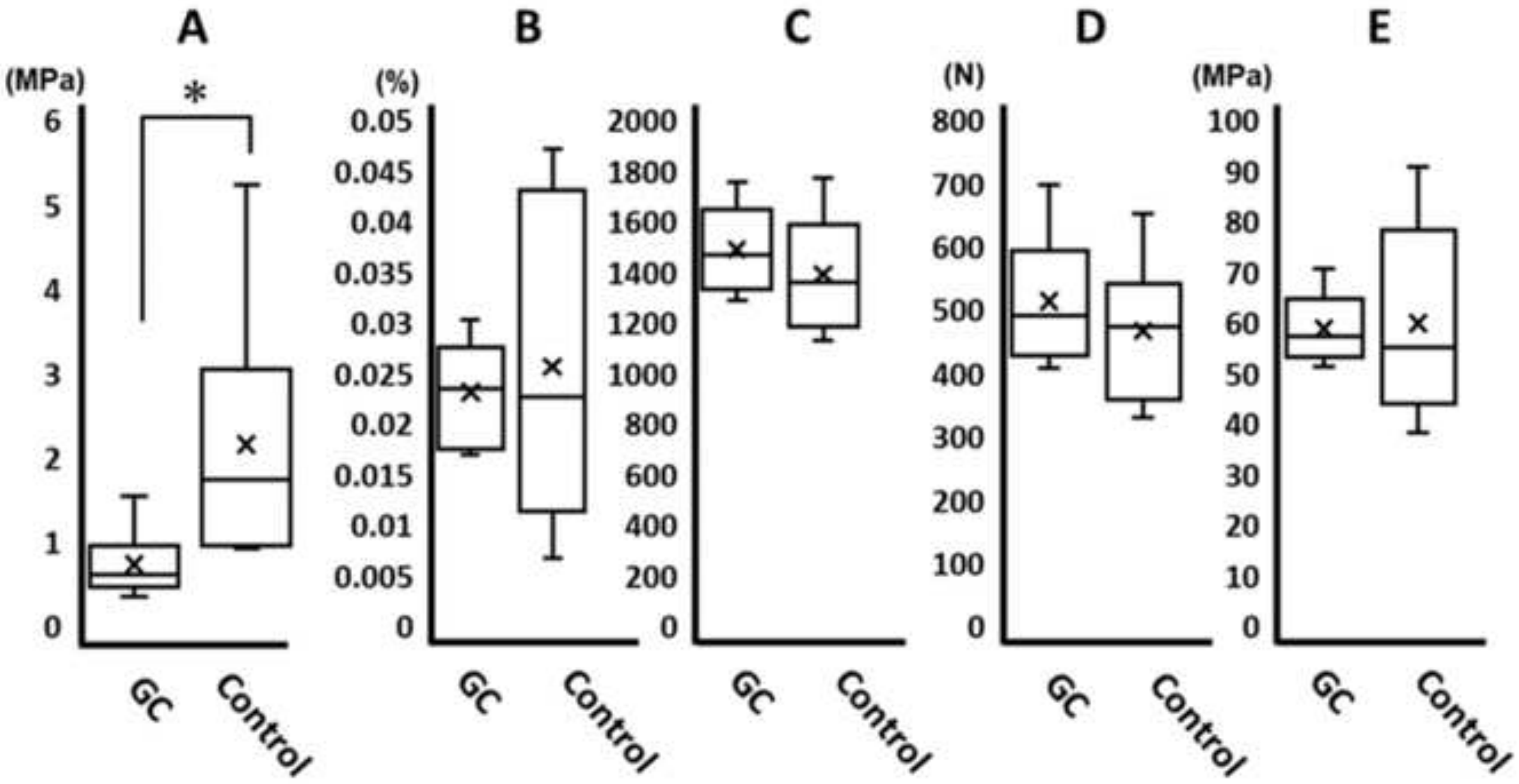

Raw data

Click here to access/download
Supporting Information
raw data.xlsx
